# Sexual reproduction in a natural *Trypanosoma cruzi* population

**DOI:** 10.1371/journal.pntd.0007392

**Published:** 2019-05-20

**Authors:** Alexander S. F. Berry, Renzo Salazar-Sánchez, Ricardo Castillo-Neyra, Katty Borrini-Mayorí, Claudia Chipana-Ramos, Melina Vargas-Maquera, Jenny Ancca-Juarez, César Náquira-Velarde, Michael Z. Levy, Dustin Brisson, the Chagas Disease Working Group in Arequipa

**Affiliations:** 1 Division of Gastroenterology, Hepatology, and Nutrition, Children’s Hospital of Philadelphia, Philadelphia, Pennsylvania, United States of America; 2 Universidad Peruana Cayetano Heredia/University of Pennsylvania Chagas Disease Field Laboratory, Arequipa, Peru; 3 Department of Biostatistics, Epidemiology and Informatics, The Perelman School of Medicine of the University of Pennsylvania, Philadelphia, Pennsylvania, United States of America; 4 Department of Biology, University of Pennsylvania, Philadelphia, Pennsylvania, United States of America; Universidade Federal de Minas Gerais, BRAZIL

## Abstract

**Background:**

Sexual reproduction provides an evolutionary advantageous mechanism that combines favorable mutations that have arisen in separate lineages into the same individual. This advantage is especially pronounced in microparasites as allelic reassortment among individuals caused by sexual reproduction promotes allelic diversity at immune evasion genes within individuals which is often essential to evade host immune systems. Despite these advantages, many eukaryotic microparasites exhibit highly-clonal population structures suggesting that genetic exchange through sexual reproduction is rare. Evidence supporting clonality is particularly convincing in the causative agent of Chagas disease, *Trypanosoma cruzi*, despite equally convincing evidence of the capacity to engage in sexual reproduction.

**Methodology/ Principle Findings:**

In the present study, we investigated two hypotheses that can reconcile the apparent contradiction between the observed clonal population structure and the capacity to engage in sexual reproduction by analyzing the genome sequences of 123 *T*. *cruzi* isolates from a natural population in Arequipa, Peru. The distribution of polymorphic markers within and among isolates provides clear evidence of the occurrence of sexual reproduction. Large genetic segments are rearranged among chromosomes due to crossing over during meiosis leading to a decay in the genetic linkage among polymorphic markers compared to the expectations from a purely asexually-reproducing population. Nevertheless, the population structure appears clonal due to a high level of inbreeding during sexual reproduction which increases homozygosity, and thus reduces diversity, within each inbreeding lineage.

**Conclusions/ Significance:**

These results effectively reconcile the apparent contradiction by demonstrating that the clonal population structure is derived not from infrequent sex in natural populations but from high levels of inbreeding. We discuss epidemiological consequences of this reproductive strategy on genome evolution, population structure, and phenotypic diversity of this medically important parasite.

## Author summary

The rearrangement of alleles among individuals in a population during sexual reproduction maintains high allelic diversity within individuals in a population at polymorphic genes. Allelic diversity within individuals can be particularly important for parasites as it enhances their ability to evade host immune systems. Despite the potential benefits of sexual reproduction for parasites, natural populations of the protozoan parasite—and causative agent of human Chagas disease—*Trypanosoma cruzi*, exhibit clonal population structures indicative of asexual reproduction. This is particularly surprising as *T*. *cruzi* has the capacity for sexual reproduction. Here, we resolve this apparent contradiction by sequencing whole genomes of 123 *T*. *cruzi* isolates from a natural population in Arequipa, Peru. Evidence of past sexual reproduction and allelic rearrangements are common in this *T*. *cruzi* population. However, the majority of sexual reproduction events occur between close relatives resulting in an apparent clonal population structure. Sexual reproduction with distant relatives in areas with greater strain diversity has the potential to affect public health by increasing diversity in immune evasion genes within individuals and enhancing within-host survival, rapidly diversifying antigens that could affect the sensitivity of serological diagnostics, and by generating diversity in pathogenicity or drug resistance.

## Introduction

**Fig 1:**
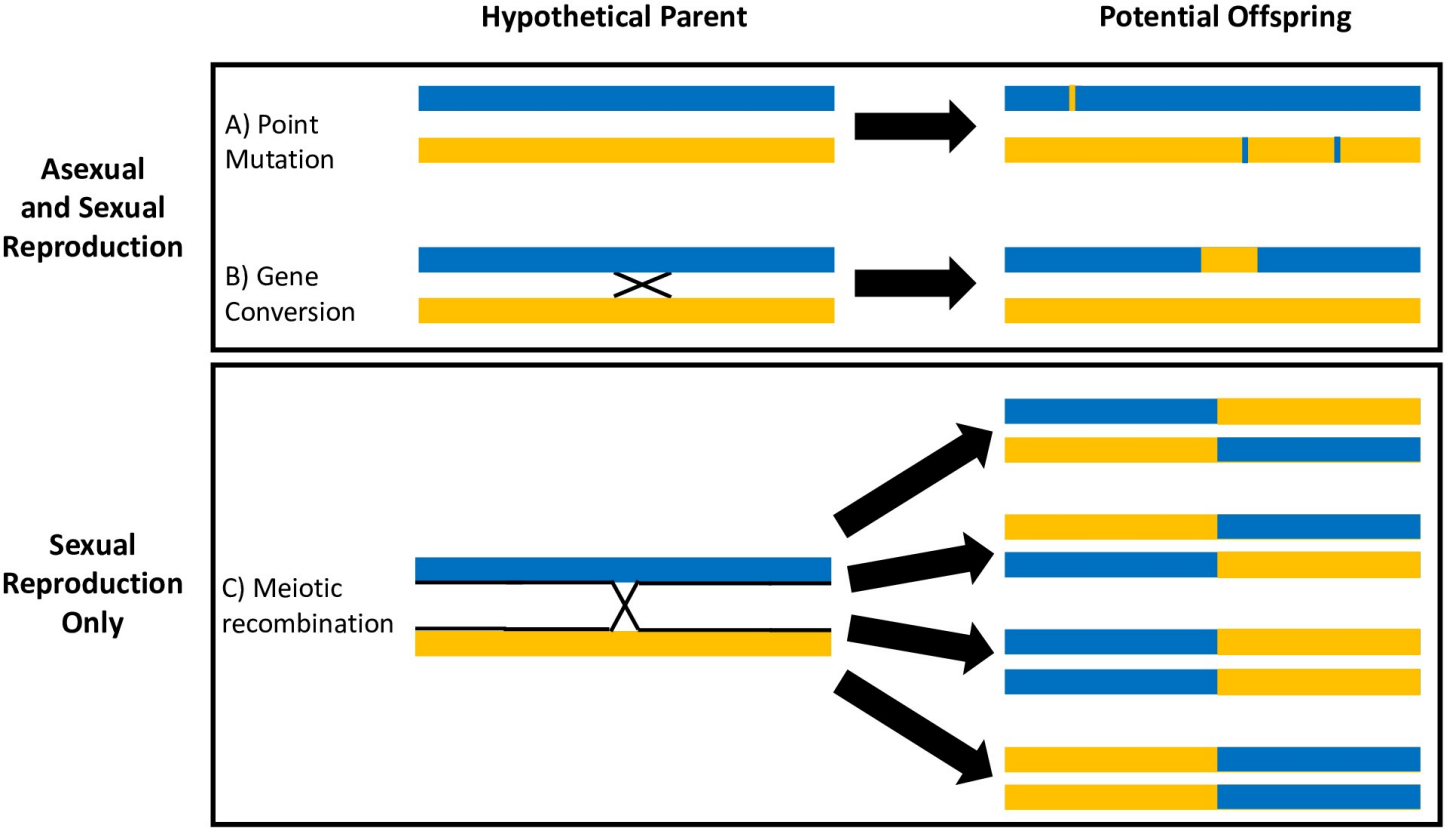
Reproductive strategies can be inferred from the distribution of polymorphic markers within and among strains in a population. Both asexual and sexual populations experience both A) point mutations and B) gene conversion events while only populations reproducing sexually experience C) meiotic recombination that reassort large genomic regions among chromosomes as well as chromosomes among individuals. **A)** Point mutations alter random bases individually such that spatial clustering of mutations is not expected in most cases. Whole chromosomes carrying novel point mutations, along with all linked markers, will be passed to offspring during asexual reproduction. These markers can disassociate due to cross-over events and independent assortment of chromosomes during meiosis and sexual reproduction. **B)** Gene conversion events homogenize small regions of homologous chromosomes (<10kb), effectively reducing diversity at multiple markers in one chromosomal region. **C)** Sexual reproduction can result in the disassociation of polymorphic markers due to the independent assortment of chromosomes as well as crossing over between homologous chromosomes during meiosis. Crossing over results in the exchange of large chromosomal segments (up to Mbs) among homologous chromosomes, disrupting associations among polymorphic markers on each chromosome. Crossing over thus results in gametes that contain chromosomes comprised of a set of polymorphic sites derived from the one parental chromosome and a set derived from the other parental chromosome. As only a small number of cross-over events are expected to occur on each chromosome during meiosis (~1), a small number of large genomic regions reassort on each resulting chromosome, a genetic signature that is distinct from point mutations and gene conversion. Random fusion of gametes with these recombined chromosomes will produce offspring with some chromosomal regions that are uniformly homozygous at polymorphic markers which are heterozygous in their siblings.

An increasing body of evidence suggests that many eukaryotic microparasites exhibit highly-clonal population structures [1–9]. That is, isolates sampled in nature tend to represent independently evolving, asexual lineages with limited genetic exchange among lineages. Despite increasingly detailed studies supporting the apparent rarity of sexual reproduction in many eukaryotic microparasites, this diverse group of organisms is capable of sexual reproduction and evidence of sex has been observed using population genetic methods and experimentally demonstrated in several species [10–16]. While sexual reproduction can generate untested combinations of alleles that are less successful than the parental combinations on average (recombinational load [17]), sex is also expected to provide several evolutionary advantages to parasites including increasing within-individual diversity in immune evasion genes where diversity is paramount for immune evasion and thus survival. We investigated two hypotheses that could explain the apparent contradiction between the observed clonal population structure and the capacity to engage in sexual reproduction in the protozoan parasite and causative agent of Chagas disease, *Trypanosoma cruzi*. Like many microparasites, *T*. *cruzi* tends to exhibit highly clonal population structures in nature [18–20] with rare exceptions [21,22], despite experimental evidence of the capacity for sexual reproduction [23]. In the first hypothesis, meiosis and fertilization are exceedingly rare such that mutational processes occur much more frequently than meiosis (Fig 1) and in the second, sexual reproduction is common but occurs between closely-related individuals resulting in the high homozygosity across genomes observed in natural populations.

The clonal population structure of *T*. *cruzi* is well-documented leading many to accept that this parasite reproduces asexually despite its capacity for sexual reproduction [5]. Asexual reproduction results in an excess of homozygosity compared to sexual populations as well as non-random associations of alleles across loci (linkage disequilibrium), both of which have been observed in natural *T*. *cruzi* populations [18–20]. For example, Oliveira *et al*. 1998 [18] found that homozygosity at, and linkage disequilibrium between, eight microsatellite loci among 24 *T*. *cruzi* strains was significantly greater than expected assuming random mating, suggesting a limited role for sexual reproduction [5]. Nevertheless, the capacity to engage in sexual reproduction in *T*. *cruzi* has been demonstrated in controlled experiments where clones transfected with different markers were mixed *in vitro* and progeny harboring both markers were observed [23]. Additionally, hybrid clones have been isolated from natural populations that formed through sexual reproduction between distantly-related lineages [24–28].

The demonstrated capacity of *T*. *cruzi* to reproduce sexually along with the potential advantages of sex is remarkable given that the population genetic patterns associated with sex (Fig 1) are not observed in natural populations [18–20]. That is, regular sexual reproduction randomly combines alleles at each locus resulting in the proportions of heterozygotes and homozygotes that conform to Hardy-Weinberg expectations, a result that has not been observed in any *T*. *cruzi* population investigated [18–20]. Similarly, sexual reproduction is expected to break down allelic associations among loci due to the independent assortment of chromosomes as well as cross-over events that occur during meiosis. By contrast, empirical studies demonstrate high levels of linkage disequilibrium among alleles in natural populations [18,29,30]. In the present study, we investigated the pattern of genomic signatures associated with the combinations of allelic markers expected in sexual and asexual populations to investigate the potential causes of the apparent contradiction between the observed clonal population structure and the capacity to engage in sexual reproduction.

## Results

**Fig 2:**
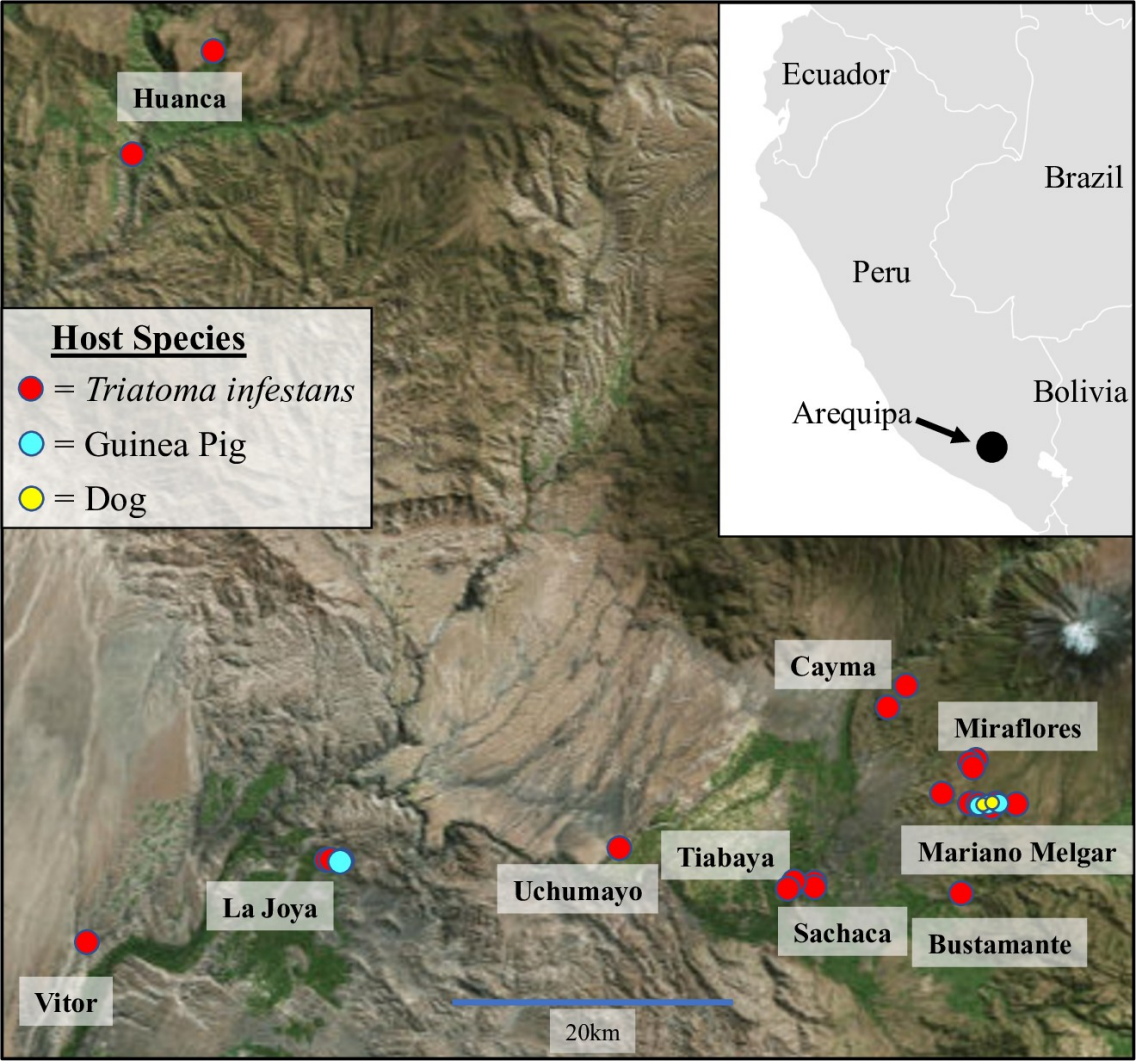
Geographic distribution of isolates. The location of all 123 sampled *T*. *cruzi* are represented by dots and colored by the host species. Inset shows the location of Arequipa. Map of Arequipa was obtained from https://landlook.usgs.gov and was modified using QGIS v. 2.18 [69]. Inset was modified from https://commons.wikimedia.org/wiki/Atlas_of_the_world.

The genome sequences from 123 *T*. *cruzi* isolates collected throughout Arequipa, Peru (Fig 2) were highly similar, suggesting that all isolates descended from a recent common ancestor. The 15,357bp maxicircle was nearly identical among isolates with an average pairwise difference of 1.04 [31] and only 12,256 sites in the ~56Mbp diploid genome were polymorphic. There is no evidence of introgression of chromosomes or segments of chromosomes into this population from outside populations as all runs of polymorphic markers are distributed throughout the phylogeny of these 123 isolates. Additionally, there are no runs of polymorphic markers that are similar to any of the outgroup genome sequences. These data suggest that the common ancestor of all sampled individuals is relatively recent and contained much of the variation observed in the isolates sequenced.

**Fig 3:**
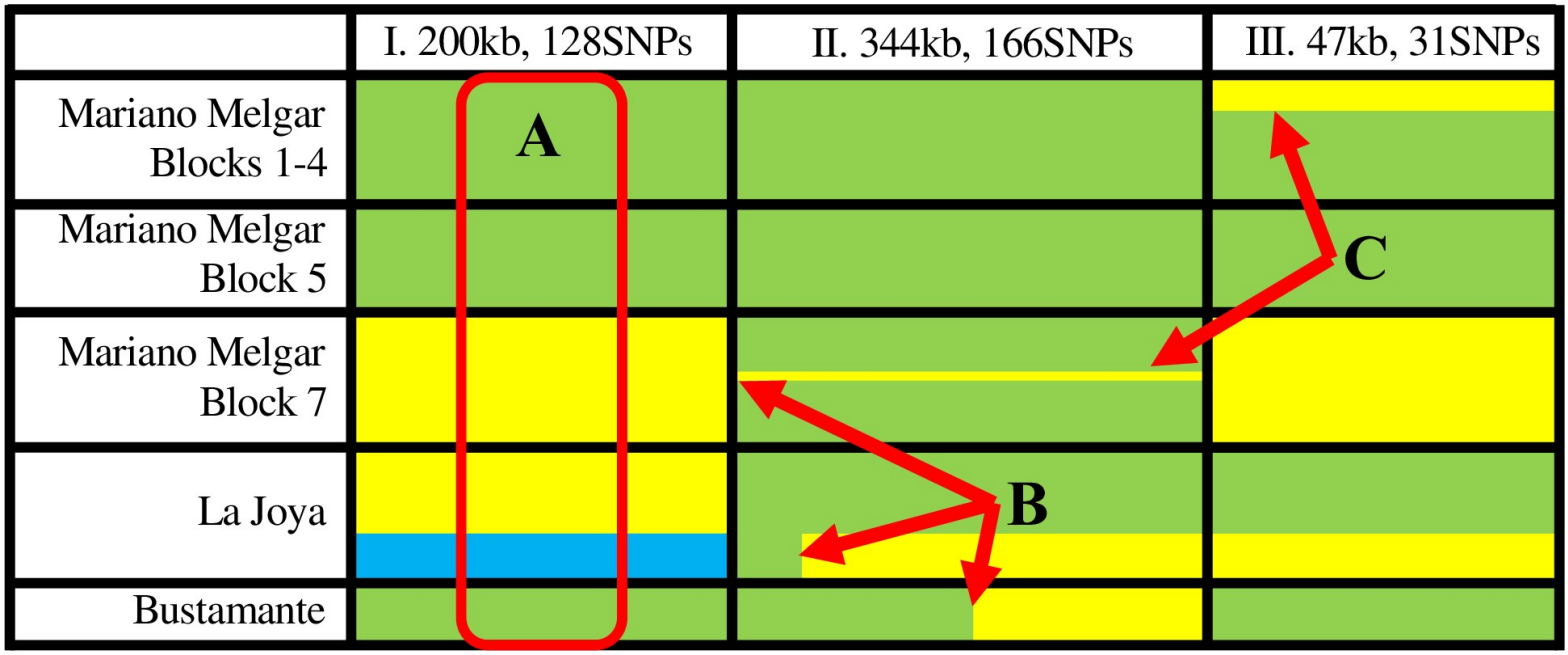
The distribution of polymorphic markers within and among individuals provides clear evidence of meiotic recombination. This representative subset of the data portrays the distributions of 325 polymorphic markers throughout three regions of the genome in 54 isolates (grouped by geographic location: Mariano Melgar Blocks 1–4 (N = 14), Block 5 (N = 11), Block 7 (N = 14), La Joya (N = 14), and Bustamante (N = 1)) that indicate the presence of sexual reproduction as A) the independent assortment of chromosomes among individuals in a population; B) meiotic recombination events that reassort polymorphic markers along a chromosome; and C) evidence of very recent reassortment of polymorphic markers via sexual reproduction. Markers that are polymorphic in the population can be heterozygous (green) or homozygous in each individual (yellow representing a site that is homozygous for one base, blue representing individuals homozygous for the alternative base). For clarity, only polymorphic sites with a minor allele frequency >16% in the population are shown here. **(A)** Independent assortment of chromosomes and subsequent fusion of gametes has resulted in a region (I) that is homozygous in some individuals and heterozygous in others across a 200kb region containing 128 polymorphic markers on an 829kb contig. In this region, individuals can be homozygous for either set of linked polymorphic markers. Interestingly, genotypes are geographically clustered, indicating identity by descent. Runs of identical color indicate identical sequence. **(B)** Meiotic recombination has resulted in a region (II) of a 357kb contig that partially retain the linkage patterns of both parental chromosomes while the rest retains the linkage pattern of only one parental chromosome, showing how crossing over can affect parts of chromosomes. In this region, there is evidence of at least three independent meiotic recombination events. (**C)** Sexual reproduction after a recent colonization of a city block has resulted in the genetic divergence of closely related samples over a run of polymorphic markers. For example, a meiotic recombination event is apparent in region II in one sampled individual on Block 7. The remaining contigs (S1 Fig) contain near-complete sequence similarity among all clones isolated from Block 7 suggesting that all individuals on this block share a recent common ancestor and the meiotic event occurring in region II occurred after the Block 7 was colonized. Similarly, a meiotic recombination event in a region (III) of a 130kb contig occurred in one lineage inhabiting Blocks 1–3 in Mariano Melgar. The regions shown here contain only some of the 474 meiotic recombination events distributed across 151 contigs, with each detected meiotic recombination event spanning from 10kb to nearly 500kb. The relative positions of each contig within the genome are unknown. For data spanning the whole genome, see S1 Fig.

Evidence of sexual reproduction was inferred from the distribution of polymorphic markers within and among sequenced isolates. The exchange of genetic material between non-sister chromatids of homologous chromosomes during meiosis results in gametes that contain chromosomes comprised of a set of polymorphic markers derived from one parental chromosome and a set derived from the other parental chromosome. When these gametes fuse and produce diploid offspring, some offspring will have regions of their chromosomes that are uniformly homozygous at the polymorphic markers while the same region will be heterozygous in other offspring (Fig 1C). Evidence of sexual reproduction was detected by identifying large segments (>10kb) of chromosomes where all of the markers (≥20) that are heterozygous in some isolates are homozygous in other isolates within the same population. Such linkage blocks containing loci in high linkage disequilibrium are common in the *T*. *cruzi* genomes in Arequipa (Fig 3; S1 Fig). Using a conservative algorithm to detect chromosomal segments derived from meiotic recombination, we identified 474 recombined segments ranging in size from 10kb to 468kb (S2 Fig). Evidence of meiotic recombination events were detected on 69 of the 80 (86.25%) contigs longer than 100kb, suggesting that this phenomenon occurs throughout the *T*. *cruzi* genome.

The conservative criteria used to identify these meiotic recombination events are unlikely to be met on contigs shorter than 100kb (S3 Fig). Reassortment of chromosomal segments through sexual reproduction (meiotic recombination or independent assortment of chromosomes) accounts for 95% of the average pairwise differences among *T*. *cruzi* isolates, with the remaining differences explained by point mutations.

Isolates derived from geographically-proximal locations commonly have the same recombination events, suggesting identity-by-descent (S4 and S5 Figs). Samples collected in the same year or from the same host, as opposed to collections from geographically similar areas, do not form monophyletic clades (S5 Fig). These data suggest that geography is the most important predictor of phylogenetic relationship.

The frequency of heterozygotes in this population is higher than expected from a Hardy-Weinberg population at 95% of polymorphic markers while only 0.10% of markers have more homozygotes than expected. Of those markers in Hardy-Weinberg equilibrium, only one (0.2%) site had a minor allele frequency greater than 0.1. Of the ten markers with more homozygotes than expected, only 1 (10%) had a minor allele frequency greater than 0.1. All polymorphic markers contain only two character states.

## Discussion

The evolutionary advantages of sex for eukaryotic microparasites are numerous and include the potential to increase the diversity of immune evasion genes within individuals where diversity is paramount for survival within hosts. Yet many populations of eukaryotic microparasites that have the capacity for sexual reproduction exhibit a clonal population structure suggesting that sexual reproduction either does not occur with sufficient regularity to disrupt clonal population structures in nature or that sexual reproduction occurs primarily among closely related individuals. The genome sequence data analyzed here supports the hypothesis that *T*. *cruzi*, a eukaryotic microparasite in which clonal population structures are commonly observed despite the demonstrated capacity for sexual reproduction, engages in sexual reproduction with high levels of inbreeding. The clonal population structure observed in this and other *T*. *cruzi* populations is mostly likely accounted for by periodic meiosis and the fusion of gametes with those of closely related individuals.

The meiotic processes necessary for gamete formation and sexual reproduction create novel associations of polymorphic markers through recombination between homologous chromosomes. Fusion of a gamete containing a recombined chromosome with a gamete from the same individual containing one of the parental chromosomes during sexual reproduction will result in homozygosity throughout one large region of the chromosome while the sites that were heterozygous in the parent will remain heterozygous in the offspring in the other chromosomal region. Here, we looked for evidence of recombined chromosomes derived from meiotic processes to detect evidence of sexual recombination. Among the genome sequences from 123 *T*. *cruzi* isolates collected in Arequipa, Peru, 474 independent meiotic recombination events were identified where all of the markers (≥20) that are heterozygous in some isolates are homozygous in other isolates across a large chromosomal segment (>10kb). Sexual reproduction occurs with sufficient regularity to observe meiotic recombination events within subpopulations that have only recently established in blocks within the city (Fig 3C). These types of meiotic recombination events are present throughout the *T*. *cruzi* genome and range in size from 10kb to 468kb. The distributions of polymorphic markers observed across large segments of DNA is substantially more likely to result from meiotic recombination than identical mutations recurring in multiple lineages or through gene conversion events which impact shorter chromosomal regions (<10kb).

The population structure of *T*. *cruzi* appears clonal despite the occurrence of sexual reproduction in this natural population due to both the irregularity of sexual reproduction and the high level of inbreeding. Despite evidence of multiple meiosis and fertilization events in this relatively young population, asexual reproduction remains the dominant form of *T*. *cruzi* reproduction. The dominance of asexual reproduction has maintained both the higher than expected levels of heterozygosity observed at the majority of polymorphic markers as well as the high levels of linkage disequilibrium among the polymorphic markers within the observed linkage blocks on many contigs. In agreement with disproportionate asexual reproduction, heterozygote frequencies in this population are higher than expected from a randomly-mating sexual population in 95% of polymorphic markers. Although heterozygosity is expected to decay with repeated inbreeding, the limited number of sexual reproduction events experienced in this population since the heterozygous common ancestor have resulted in only a moderate reduction in heterozygosity among the extant descendants. The geographic structure of this population, where subpopulations cluster within city blocks (S4 and S5 Figs), may prevent gene flow, and thus recombination, between subpopulations in different geographic regions [22]. This phenomenon, known as the Wahlund effect [32], is expected to cause deviations from the expected Hardy-Weinberg equilibrium and level of linkage disequilibrium, even when sexual recombination is occurring within geographically-isolated subpopulations. These data also suggest that the common ancestor of all sampled *T*. *cruzi* genomes contained nearly all of the polymorphisms analyzed, which is also supported by data showing that most (>95%) of the genetic diversity among isolates results from the reassortment of a set of ancestral polymorphic markers. While the data suggest that the common ancestor was likely heterozygous at most polymorphic sites identified here, heterozygous sites likely comprised a very small percentage of the ancestral genome because less than 0.1% of sites are polymorphic in this population.

Variation in the number of chromosomes within each *T*. *cruzi* strain, or aneuploidy [23,33], can hinder population genomic analyses. Although the proportion of base counts derived from sequencing depth across heterozygous sites suggests this population is primarily diploid (S6 Fig), the conclusions presented here regarding the number and location of sexual recombination events observed are robust to variable ploidy levels. That is, many contigs contain internal recombination breakpoints that are detected in multiple isolates (Fig 3B), suggesting that these chromosomes have experienced a meiotic recombination event, regardless of the number of other chromosomes in the strains where these contigs were identified.

**Fig 4:**
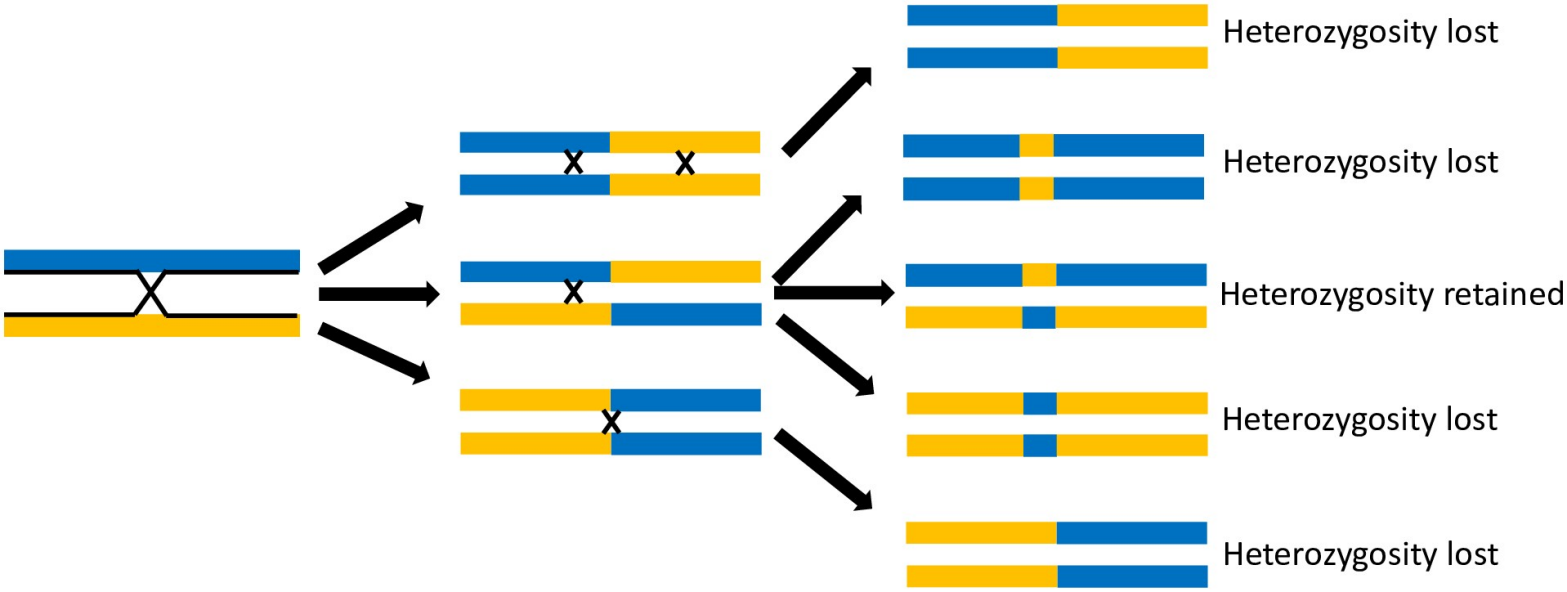
Inbreeding results in a decay of heterozygosity. After one generation of inbreeding, half of the offspring retain the heterozygosity found in the parent while half become homozygous. As diversity cannot be restored in homozygous regions in the absence of outcrossing and heterozygosity decays by half each generation, population-level heterozygosity continually declines in inbreeding populations until all lineages are homozygous.

High levels of inbreeding also cause the population structure of this *T*. *cruzi* population to appear clonal. Inbreeding limits the exchange of diverse alleles among individuals within the population resulting in multiple independently evolving lineages. Further, inbreeding reduces the benefits of sexual reproduction as it results in the continuous decay of diversity within each lineage until the absorbing boundary of complete homozygosity is attained (Fig 4). That is, only half of the offspring that result from self-mating, where gametes fuse only with gametes of the same parent, in a heterozygote lineage will be heterozygous while all offspring of homozygotes would be homozygous, thus reducing the proportion of heterozygotes in the population by half with each generation. No evidence of outcrossing was detected in the current dataset (Fig 3A). It is unclear, however, if the apparent inbreeding results from obligatory selfing or if outbreeding occurs but cannot be observed due to the limited genome-wide diversity in Arequipa. The obligatory selfing hypothesis is supported by published investigations in *T*. *cruzi* populations with greater diversity in which multiple *T*. *cruzi* lineages are frequently found in the same host [34] and vector [18,35] without evidence of outcrossing. Future studies are necessary to assess the population or molecular mechanisms that result in high levels of selfing in areas with opportunities for outcrossing.

The biological mechanism of sexual reproduction in *T*. *cruzi* may restrict outcrossing. The location (in what host or host tissue), frequency, and mechanism affecting the probability of outcrossing in *T*. *cruzi* have not been elucidated, although it is known that *Trypanosoma brucei* produces gametes in its vector [12]. By contrast, experimental evidence suggests that *T*. *cruzi* undergoes recombination in the mammalian host [23]. Haploid gametes have not yet been detected in *T*. *cruzi*, potentially due to the practical challenges of isolating an ephemeral stage of a relatively rare organism, or that *T*. *cruzi* recombination may occur via parasexual recombination. Parasexual reproduction, which occurs in several eukaryotic microbes, is the creation of a tetraploid cell through the fusion of diploid cells, followed by chromosomal crossing over during mitosis and stochastic chromosome loss to return to a diploid state [23,36,37]. The data presented here are broadly consistent with both parasexual recombination and meiosis as both mechanisms result in the exchange of large genomic segments [36]. However, the stochastic chromosome loss that occurs during parasexual reproduction is expected to generate considerable aneuploidy which was not detected in the current dataset (S6 Fig).

Sexual reproduction in parasites like *T*. *cruzi* may be particularly important for maintaining a diverse assortment of immune evasion genes within individuals [38,39]. The strong selection pressure generated by the host immune system has resulted in the extensive and diverse immune evasion gene family in *T*. *cruzi*, the trans-sialidase genes [27,40,41]. Sexual reproduction can maintain immune evasion gene diversity in the face of homogenization events that commonly occur among paralogs within genomes by redistributing variation among trans-sialidase genes within a population through chromosome sorting or cross-over events [42–44]. While we did not specifically investigate trans-sialidase genes, it is possible that even in populations where inbreeding is common, low frequencies of sex can increase trans-sialidase diversity within individuals, thus increasing the probability of survival within a host. Future studies could assess whether recombination hotspots are conserved across *T*. *cruzi* populations and if recombination events impact the diversity of trans-sialidase genes.

Since the *T*. *cruzi* isolates used in this analysis were maintained in culture for variable periods of time, meiosis could have occurred during the isolation and culturing process. However, 65% (310) of all crossing over events were present in multiple samples. Thus, a substantial proportion of the crossing over detected here must have occurred prior to isolation.

Although the ubiquitous observations of clonal population structures have resulted in the hypothesis that the frequency of sexual reproduction is insufficient to disrupt the clonal population structure in natural *T*. *cruzi* populations [25,45], the data presented here suggest that sexual recombination is not uncommon in natural populations. Sexual reproduction occurred repeatedly in the recent evolutionary history of this population and has occurred within subpopulations that have only recently established on city blocks (Fig 3C). The number of meiotic recombination events reported here is conservative, as events occurring between homozygous regions cannot be detected and our criteria for identifying recombined regions was strict. Nevertheless, the extant linkage disequilibrium across large linkage blocks suggests that asexual reproduction has been the most common mode of reproduction in this population. This mixed life-history strategy has important medical and evolutionary implications. Sexual reproduction may allow for rapid diversification of antigens which may contribute to the variability in serological diagnostics [46–48] and has the potential to generate genetic and phenotypic diversity [49,50] in pathogenicity [51,52], host and vector propensity [53,54], and vulnerability to drugs [55,56] if outcrossing is common in populations with greater diversity. Primarily asexual reproduction coupled with periodic sexual inbreeding, on the other hand, will result in clonal population structures that maximize the diversity among lineages and minimize the possibility that virulence factors or drug resistance will introgress into other lineages. Primarily asexual reproduction coupled with periodic sexual outcrossing may be significant for the adaptive evolution in novel environments and may be particularly important for invading urbanizing environments where rapid adaptation may be at a premium.

## Materials and methods

### Ethics Statement

The Institutional Animal Care and Use Committee (IACUC) of Universidad Peruana Cayetano Heredia reviewed and approved the animal-handling protocol used for this study (identification number 59605). The IACUC of Universidad Peruana Cayetano Heredia is registered in the National Institutes of Health at the United States of America with PHS Approved Animal Welfare Assurance Number A5146-01 and adheres to the Animal Welfare Act of 1990 [57].

### Sample collection and study site

Genomic DNA from 123 *T*. *cruzi* isolates was sequenced to uncover evidence of sexual reproduction (Fig 2). Samples were isolated from *Triatoma infestans* bugs collected in Arequipa, Peru (N = 114) and an additional nine samples were isolated from the blood of guinea pigs (N = 7) and dogs (N = 2) in Arequipa (S1 Table). Uninfected *T*. *infestans* were allowed to feed on six of the nine blood samples to in order to transfer the parasite to a natural vector. For all samples (lab- and naturally-infected vectors), *T*. *cruzi* was isolated by injecting feces from infected vectors into guinea pigs or mice and re-isolating *T*. *cruzi* from the blood of each infected mammal as previously described [58]. *T*. *cruzi* was isolated directly from the blood samples of three guinea pigs collected in Arequipa without passage through *T*. *infestans*.

### Sequencing

DNA from all laboratory cultures was extracted using Qiagen DNEasy DNA Purification Kit. 150bp single-end read libraries were prepared using TruSeq Nano kit and sequenced to an average depth of ~50X using Illumina’s NextSeq500 (reads available at Sequence Read Archive (SRA) under the BioProject PRJNA517719). Six *T*. *cruzi* isolates were prepared in duplicate, and one in triplicate, to allow estimation of sequencing error. Low quality bases were trimmed from raw reads using trimmomatic-0.32 [59].

### Whole genome assembly

Phylogenetic analyses demonstrate that the reference genome most closely related to the Arequipan isolates is the discrete typing unit 1 (DTU1) genome TcJR clone 4 (S7 Fig). Arequipan genomes were assembled using the TcJR clone 4 genome, obtained from TriTrypDB (http://tritrypdb.org/tritrypdb/), as a reference using bowtie2 [60]. Only the 333 contigs longer than 10kb were used for the assembly to avoid spurious alignments to short contigs, for a total genome assembly that includes nearly 28Mbp (S2 Table). This assembly largely excluded the extensive repeat regions found throughout the *T*. *cruzi* genome. Duplicate reads were removed using Picard MarkDuplicates [61].

### SNP calling

Individual gVCF files containing SNP data for each sample were generated using GATK HaplotypeCaller [61,62] following GATK’s Best Practices procedure [63,64]. A joint genotype file containing all polymorphic sites from all samples was created using GATK GenotypeGVCF. Indels were excluded. Polymorphic loci were hard-filtered by quality using GATK VariantFiltration, requiring Fisher strand bias (FS) <40, mapping quality (MQ) >30, and quality by depth (QD) >10. Only loci for which all samples achieved a minimum depth of 20 and a Genotype Quality score (GQ) greater than 40 were included. These filters maximized the number of polymorphic sites identified while ensuring that duplicate and triplicate sequences resulted in identical SNP datasets. The final consensus SNP panel included 9271 polymorphic sites. To assess ploidy in these isolates, the proportion of reads containing each nucleotide was calculated for all heterozygous sites in all 123 isolates.

### Detection of meiotic recombination events

Cross-over events during meiosis create a novel combination of polymorphic markers by combining large genetic regions of homologous chromosomes (Fig 1C). During meiosis, non-sister chromatids of homologous chromosomes can exchange genetic material resulting in gametes containing chromosomes that differ from either parental chromosome. The chromosomes that result from a meiotic cross-over event in gametes are comprised of two sections, one retaining the linkage association among markers found on one of the parental chromosomes joined with another chromosomal segment with the linkage associations found on the other parental chromosome. Fusion of gametes containing chromosomes that have experienced different cross-over events will result in regions of the chromosome that are heterozygous in some diploid offspring and homozygous in others. Thus, detection of large segments (>10kb) of chromosomes where all of the polymorphic markers (>20) that are heterozygous in some isolates are homozygous in other isolates within the same population indicates that a meiotic cross-over event created a novel combination of markers. Long chromosomal regions with many (>20) polymorphic markers that are heterozygous in some isolates but homozygous in others are unlikely to result from independent point mutations due to the clustering of all of the mutational events in one region of a chromosome. Additionally, the point mutation hypothesis would necessitate that the same combination of mutations occurred independently in multiple isolates. Gene conversion could also produce the pattern expected from meiotic crossing over although gene conversion events are typically restricted to much shorter chromosomal segments (<10kb).

We examined the genomic sequence data for the distributions of polymorphic markers within and among individuals that are expected to result from meiosis. To this end, we first aligned all polymorphic sites by contig and position using VCF tools v0.1.13 [65] and PLINK [66]. The data were converted to a VCF file containing SNP data for all 123 samples and subsequently to PLINK format using VCF tools. PLINK’s runs of homozygosity function was used to generate a list of regions that were entirely homozygous in some isolates and heterozygous in at least one isolate, a pattern suggestive of a meiotic recombination event. A conservative estimate of the number and size of recombinant regions was obtained by defining a recombinant region as a run of homozygosity containing at least 20 consecutive, homozygous polymorphic markers that span at least 10kb with a density of at least 1 SNP per 50kb. Runs of homozygosity were determined by sliding a window of 25 SNPs across the genome and determining if each window position contained a run of homozygosity as defined. This method produces a conservative estimate of the number and size of recombination events because the minimum size of 20 consecutive homozygous SNPs will not identify runs of homozygosity on short contigs nor regions with low densities of polymorphic markers. Additionally, regions that have acquired point mutations will not be identified as the algorithm requires that a run of homozygosity cannot contain any heterozygous sites. Further, recombination between identical homozygous regions were not detected because runs of homozygosity contained by all samples were ignored. Runs of homozygosity shared by multiple samples were considered identical by descent based on sequence similarity (S8 Fig). Potential recombinant regions were verified using Recombination Analysis Tool (RAT) [67] (S1 Text).

### Hardy-Weinberg equilibrium

The frequency of sexual reproduction affects the proportions of heterozygotes and homozygotes in a population. While regular sexual reproduction results in proportions of heterozygotes and homozygotes that conform to Hardy-Weinberg expectations, infrequent sexual reproduction or inbreeding will result in allele frequencies that deviate from Hardy-Weinberg expectations. To determine the frequency of sex and the randomness of mating in this *T*. *cruzi* population, the χ^2^ test for Hardy-Weinberg equilibrium was performed for each polymorphic locus [68]. Briefly, for each locus, the difference between the expected genotype frequencies (*E*) and observed genotype frequencies (*O*) was squared and normalized by the expected genotype frequencies [*(O-E)*^*2*^*/E*]. Loci are assumed be in Hardy-Weinberg equilibrium if the sum of the χ^2^ values across genotypes is less than 3.841.

## Supporting information

S1 Fig Evidence of recombination is evident throughout the *T*. *cruzi* genome. Character state for each of 9271 polymorphic markers across all 123 *T*. *cruzi* isolates is shown. Each row represents a *T*. *cruzi* isolate which are grouped geographically by district and block within districts in the same order displayed in S1 Table. Columns represent polymorphic markers where heterozygous loci are represented in green, markers that are homozygous for one set of markers are represented in yellow, and markers that are homozygous for the other set of markers are represented in blue. All markers contain only two alleles. Black columns separate contigs. For visualization, the genome is divided into four sections. Evidence of recombination is exhibited throughout the genome, with telltale runs of homozygosity present in 151 contigs.
(TIF)

S2 Fig Length of meiotic recombination events. 474 runs of homozygosity that range in size from 10kb to 468kb were identified. Short recombination events are more common than long runs, however because of the short length of contigs, long runs are less likely to be found intact than short runs.
(TIF)

S3 Fig Number of recombination events per contig. Recombination events occur throughout the genome. Larger contigs generally have evidence of more recombination because the recombination event criteria are more likely to be met. Further, if crossover events occur randomly, more events are expected to occur in larger regions.
(TIF)

S4 Fig Isolates on city blocks commonly have the same recombination events indicating that some recombination events occurred after colonization of a city block. Geographic clustering of the 56 isolates collected in Mariano Melgar was assessed using 100 independent iterations of ADMIXTURE [70] for each number of genetic clusters (K, ranging from 2 to 8) assuming linkage disequilibrium until the log-likelihood increased by less than ε = 10^−4^ between iterations. The optimal alignment of the 100 iterations was calculated using CLUMPP [71]. **A.** Cross-validation scores are shown for each genetic cluster (K) averaged across 100 iterations. Standard error bars are shown for each value. K = 4 was determined to be the optimal number of genetic clusters. **B.** The proportion of each color within a bar represents the likelihood that a sample belongs to each of four genetic clusters, where each color represents a unique genetic cluster. The clustering of identical genotypes within blocks 1–4, within block 5, and within block 7 recapitulates the phylogenetic relationship among isolates (S5 Fig). A single isolate collected from Block 7 (yellow, red, and brown) is genetically distinct based on both the ADMIXTURE and phylogenetic analyses.
(TIF)

S5 Fig *T*. *cruzi* isolates in Arequipa tend to cluster geographically. The maximum likelihood phylogeny includes all 123 *T*. *cruzi* samples collected from Arequipa and was calculated using presence/absence for each of the 474 recombination events. Branches and collapsed clades are colored blue or yellow when they include at least one sample collected from a guinea pig or dog, respectively. While samples collected in the same year or from the same host do not form monophyletic clades, samples from the same city block tend to form monophyletic clades (*e*.*g*. Mariano Melgar blocks 1–4, 5, and 7), suggesting that geography is the most important predictor of phylogenetic relationship. Bootstrap support (1000 replicates) is labeled on all nodes. Terminal nodes with bootstrap support >0.70 were combined to highlight the effect of geography on phylogenetic relationship. The clade colored black contains samples from 10 districts and has poor bootstrap support (<0.70). Bold numbers within collapsed clades are the total number of samples included. The number of samples from guinea pigs (GP) and dogs are shown where applicable. Sample collection date ranges are also shown within collapsed clades. When samples representing multiple locations are contained within a single collapsed clade, the number of samples from each location is shown in parentheses.
(TIF)

S6 Fig Frequency of heterozygous sites in which reads contain the reference nucleotide suggests the isolates in this study are primarily diploid. The single mode of base calls around 50% at all heterozygous sites in all 123 genomes is indicative of diploidy. Bimodal distributions around 33% and 66% would indicate triploidy while trimodal distributions around 25%, 50%, and 75% would indicate tetraploidy. Sites where >80% of reads contained the same base were considered homozygous.
(TIF)

S7 Fig Arequipan isolates share a recent common ancestor with TCJRcl4. 41 distinct Arequipan genomes were aligned to both the TCJRcl4 reference genome and the maxicircle sequence of the Silvio reference strain. A neighbor-joining phylogenetic tree shows that all 41 genomes from both assemblies share a more recent common ancestor with TCJRcl4 than Silvio, and that the isolates are definitively discrete typing unit I. All nodes have >99% bootstrap support.
(TIF)

S8 Fig Criteria used to distinguish runs of homozygosity. Two runs of homozygosity were considered identical by descent (IBD) if they were 99% identical and had ten or fewer non-overlapping homozygous SNPs. That is, a run of homozygosity in one sample was considered identical to a run of homozygosity in another if it shared 99% sequence similarity and were of similar lengths, defined as ten or fewer non-overlapping homozygous SNPs on either end. Relaxing these assumptions did not qualitatively alter the results nor the conclusions.
(TIF)

S1 Table Collection locations and years for each *T*. *cruzi* sample. (PDF)

S2 Table Reference genome and genome assembly summary statistics. (TIF)

S1 Text Supplemental methods. (DOCX)
