# Supplementary figures and images for: Sexual reproduction in a natural Trypanosoma cruzi population
Source: PLoS Negl Trop Dis. 2019 May 20;13(5):e0007392. doi: 10.1371/journal.pntd.0007392 (PMC6544315; doi:10.1371/journal.pntd.0007392)

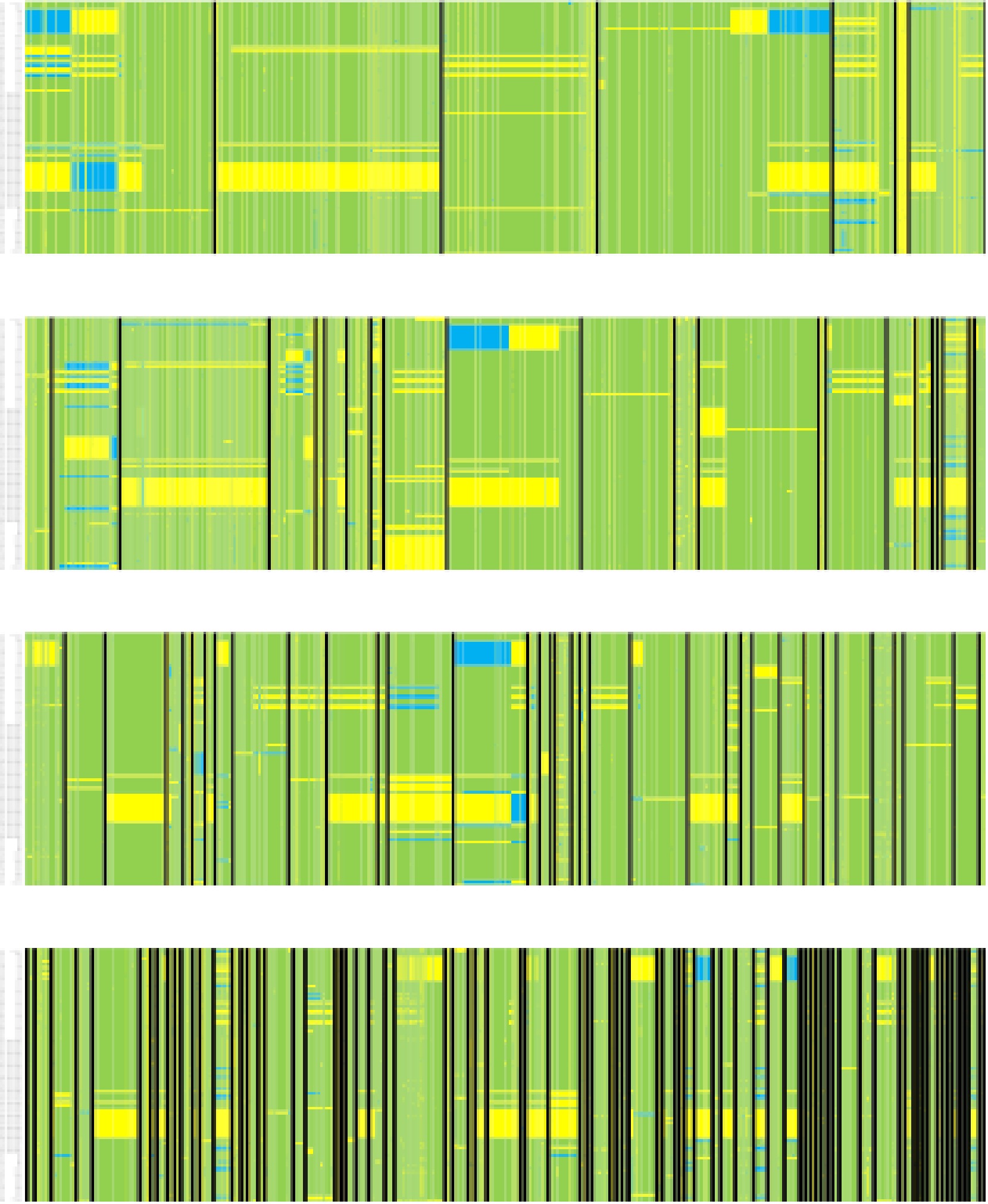

Supplement: S1 Fig — Character state for each of 9271 polymorphic markers across all 123 T. cruzi isolates is shown. Each row represents a T. cruzi isolate which are grouped geographically by district and block within districts in the same order displayed in S1 Table. Columns represent polymorphic markers where heterozygous loci are represented in green, markers that are homozygous for one set of markers are represented in yellow, and markers that are homozygous for the other set of markers are represented in blue. All markers contain only two alleles. Black columns separate contigs. For visualization, the genome is divided into four sections. Evidence of recombination is exhibited throughout the genome, with telltale runs of homozygosity present in 151 contigs. (TIF) [file pntd.0007392.s001.tif]

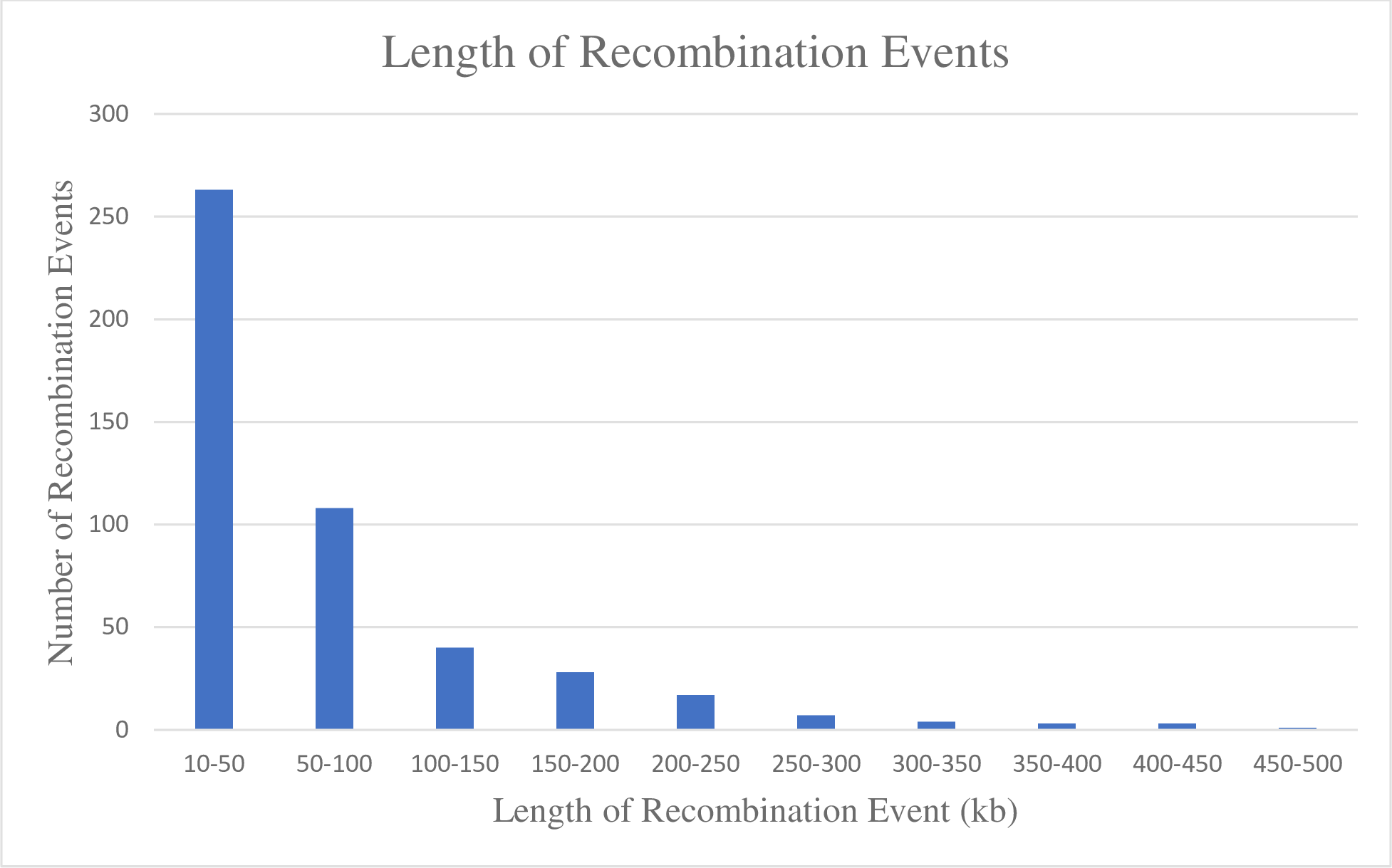

Supplement: S2 Fig — 474 runs of homozygosity that range in size from 10kb to 468kb were identified. Short recombination events are more common than long runs, however because of the short length of contigs, long runs are less likely to be found intact than short runs. (TIF) [file pntd.0007392.s002.tif]

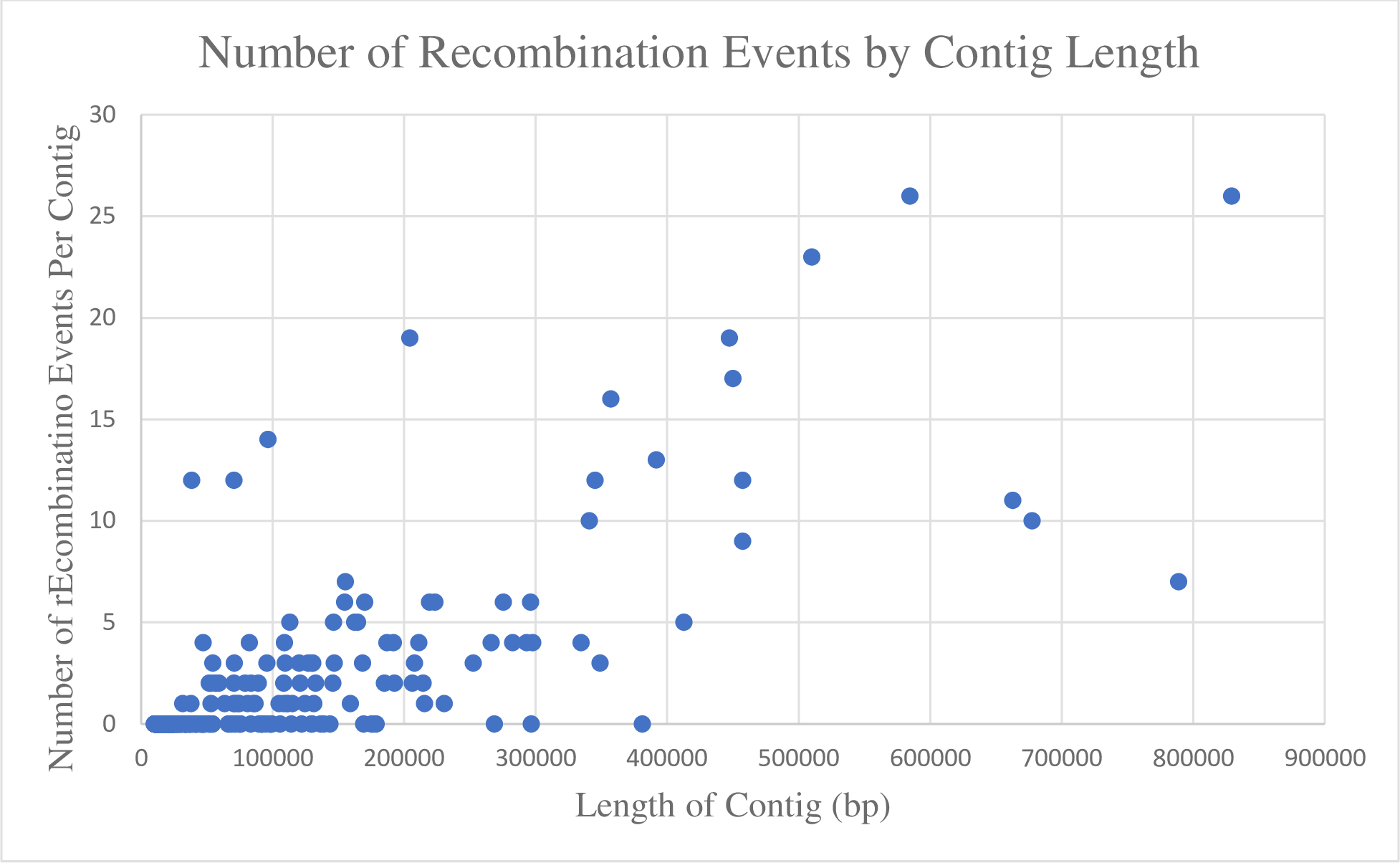

Supplement: S3 Fig — Recombination events occur throughout the genome. Larger contigs generally have evidence of more recombination because the recombination event criteria are more likely to be met. Further, if crossover events occur randomly, more events are expected to occur in larger regions. (TIF) [file pntd.0007392.s003.tif]

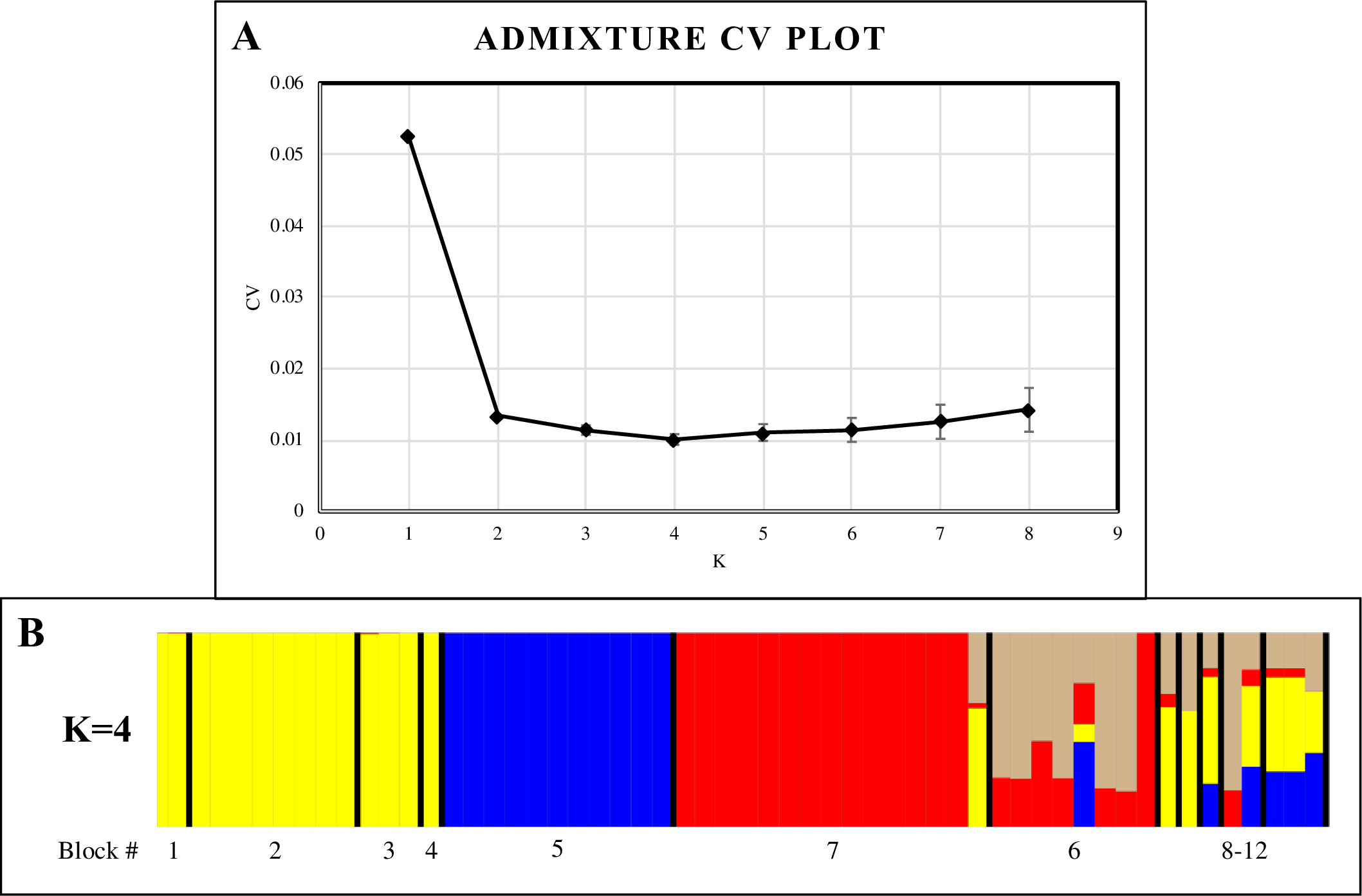

Supplement: S4 Fig — Geographic clustering of the 56 isolates collected in Mariano Melgar was assessed using 100 independent iterations of ADMIXTURE [70] for each number of genetic clusters (K, ranging from 2 to 8) assuming linkage disequilibrium until the log-likelihood increased by less than ε = 10−4 between iterations. The optimal alignment of the 100 iterations was calculated using CLUMPP [71]. A. Cross-validation scores are shown for each genetic cluster (K) averaged across 100 iterations. Standard error bars are shown for each value. K = 4 was determined to be the optimal number of genetic clusters. B. The proportion of each color within a bar represents the likelihood that a sample belongs to each of four genetic clusters, where each color represents a unique genetic cluster. The clustering of identical genotypes within blocks 1–4, within block 5, and within block 7 recapitulates the phylogenetic relationship among isolates (S5 Fig). A single isolate collected from Block 7 (yellow, red, and brown) is genetically distinct based on both the ADMIXTURE and phylogenetic analyses. (TIF) [file pntd.0007392.s004.tif]

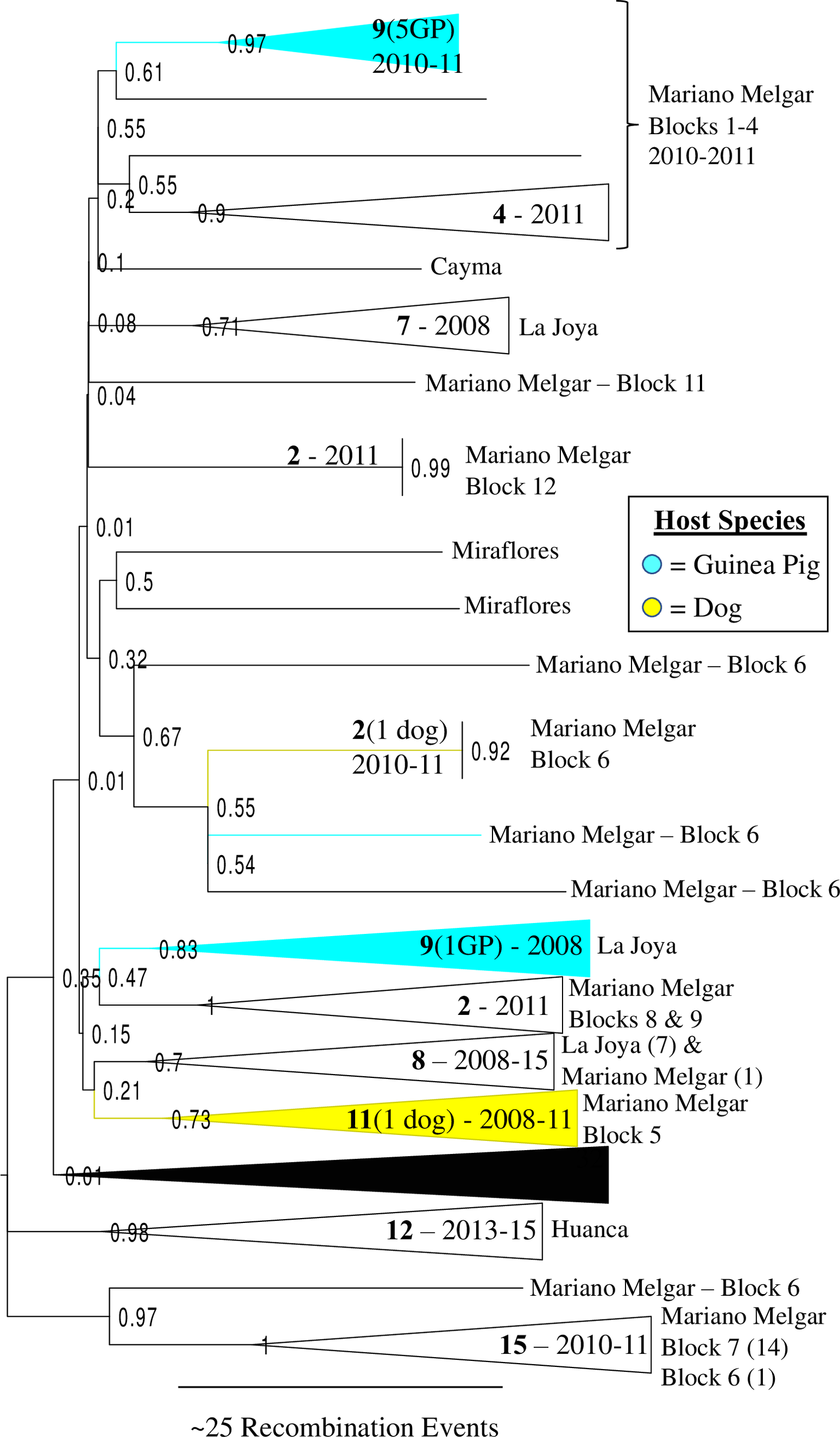

Supplement: S5 Fig — The maximum likelihood phylogeny includes all 123 T. cruzi samples collected from Arequipa and was calculated using presence/absence for each of the 474 recombination events. Branches and collapsed clades are colored blue or yellow when they include at least one sample collected from a guinea pig or dog, respectively. While samples collected in the same year or from the same host do not form monophyletic clades, samples from the same city block tend to form monophyletic clades (e.g. Mariano Melgar blocks 1–4, 5, and 7), suggesting that geography is the most important predictor of phylogenetic relationship. Bootstrap support (1000 replicates) is labeled on all nodes. Terminal nodes with bootstrap support >0.70 were combined to highlight the effect of geography on phylogenetic relationship. The clade colored black contains samples from 10 districts and has poor bootstrap support (<0.70). Bold numbers within collapsed clades are the total number of samples included. The number of samples from guinea pigs (GP) and dogs are shown where applicable. Sample collection date ranges are also shown within collapsed clades. When samples representing multiple locations are contained within a single collapsed clade, the number of samples from each location is shown in parentheses. (TIF) [file pntd.0007392.s005.tif]

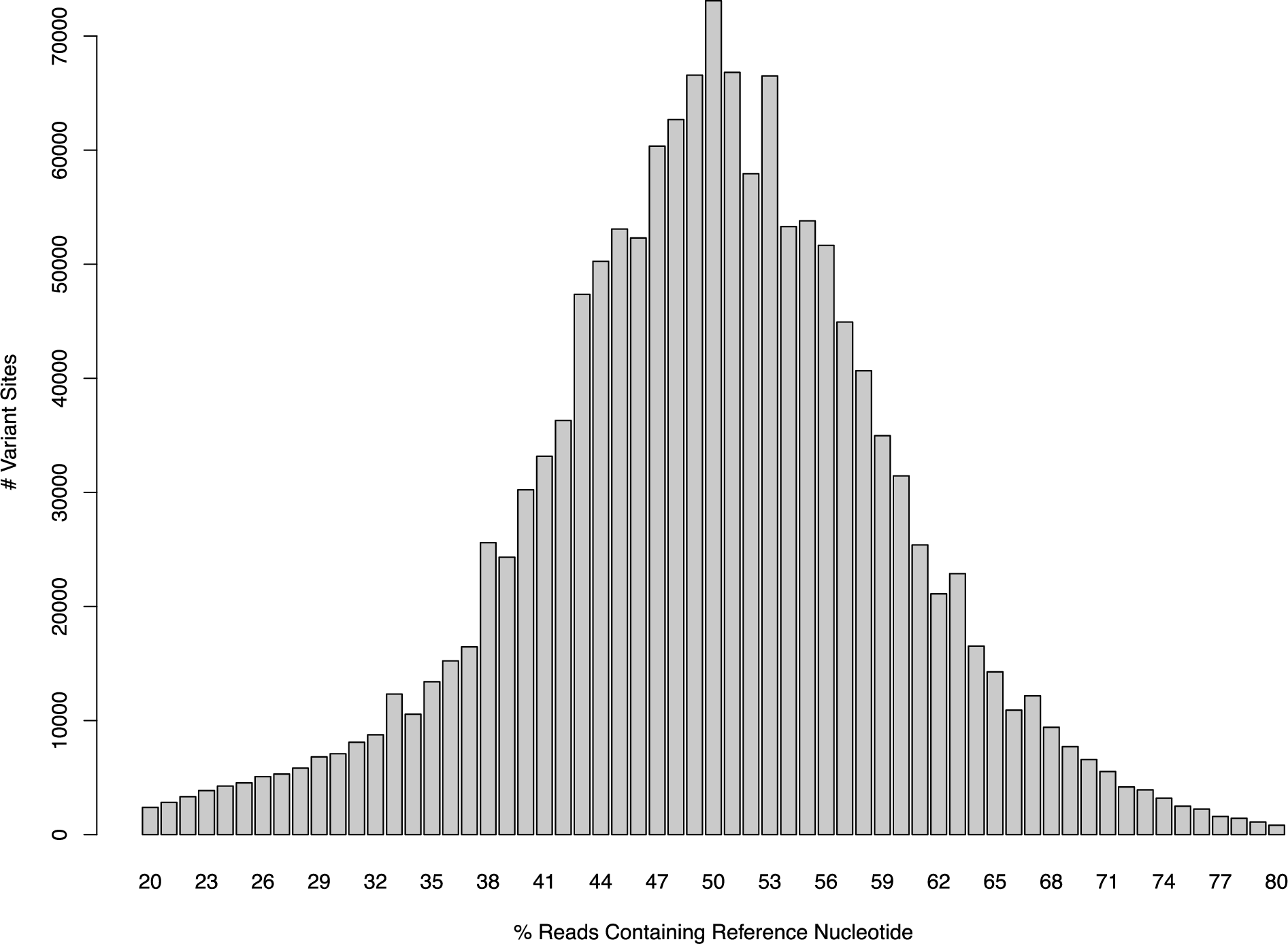

Supplement: S6 Fig — The single mode of base calls around 50% at all heterozygous sites in all 123 genomes is indicative of diploidy. Bimodal distributions around 33% and 66% would indicate triploidy while trimodal distributions around 25%, 50%, and 75% would indicate tetraploidy. Sites where >80% of reads contained the same base were considered homozygous. (TIF) [file pntd.0007392.s006.tif]

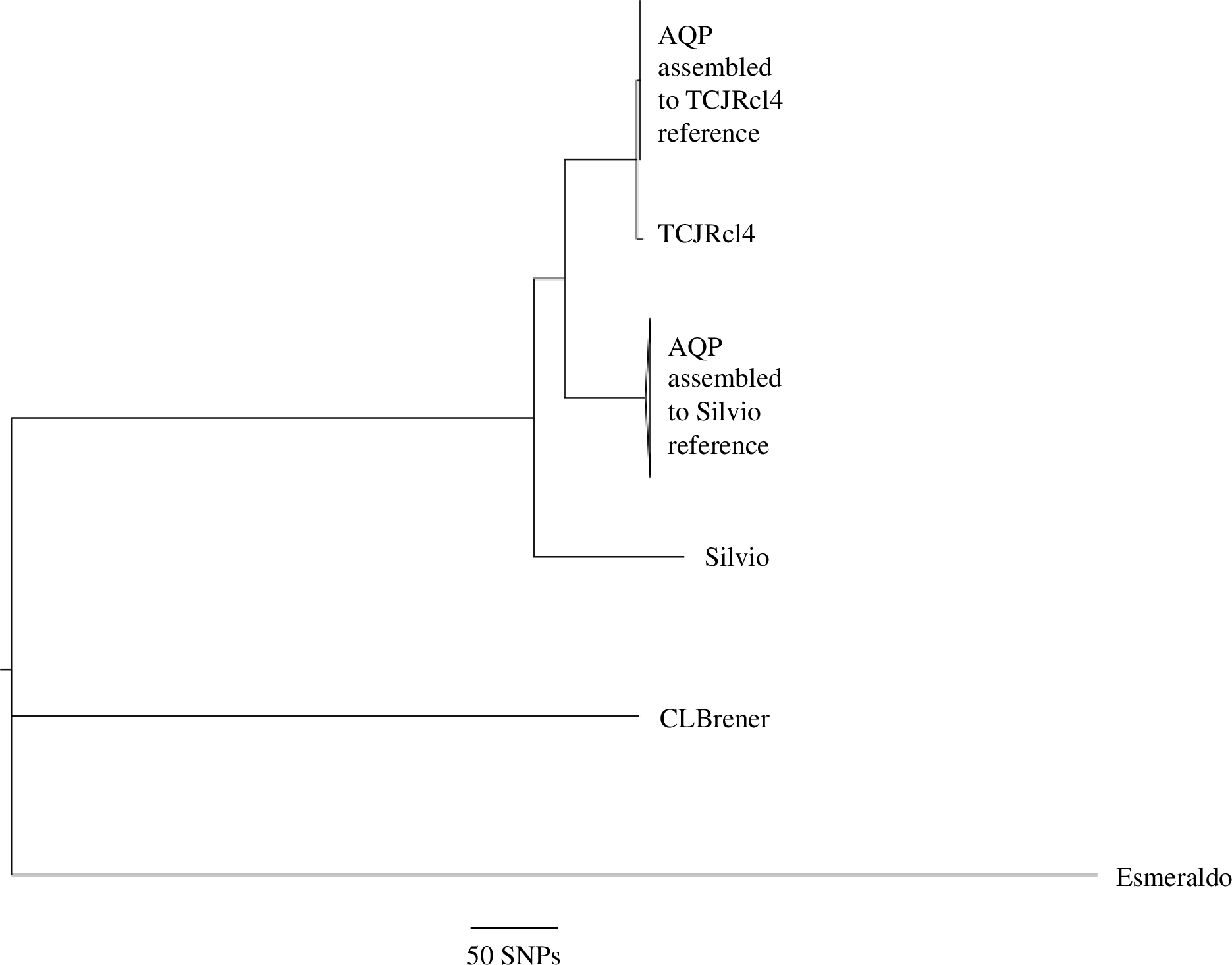

Supplement: S7 Fig — 41 distinct Arequipan genomes were aligned to both the TCJRcl4 reference genome and the maxicircle sequence of the Silvio reference strain. A neighbor-joining phylogenetic tree shows that all 41 genomes from both assemblies share a more recent common ancestor with TCJRcl4 than Silvio, and that the isolates are definitively discrete typing unit I. All nodes have >99% bootstrap support. (TIF) [file pntd.0007392.s007.tif]

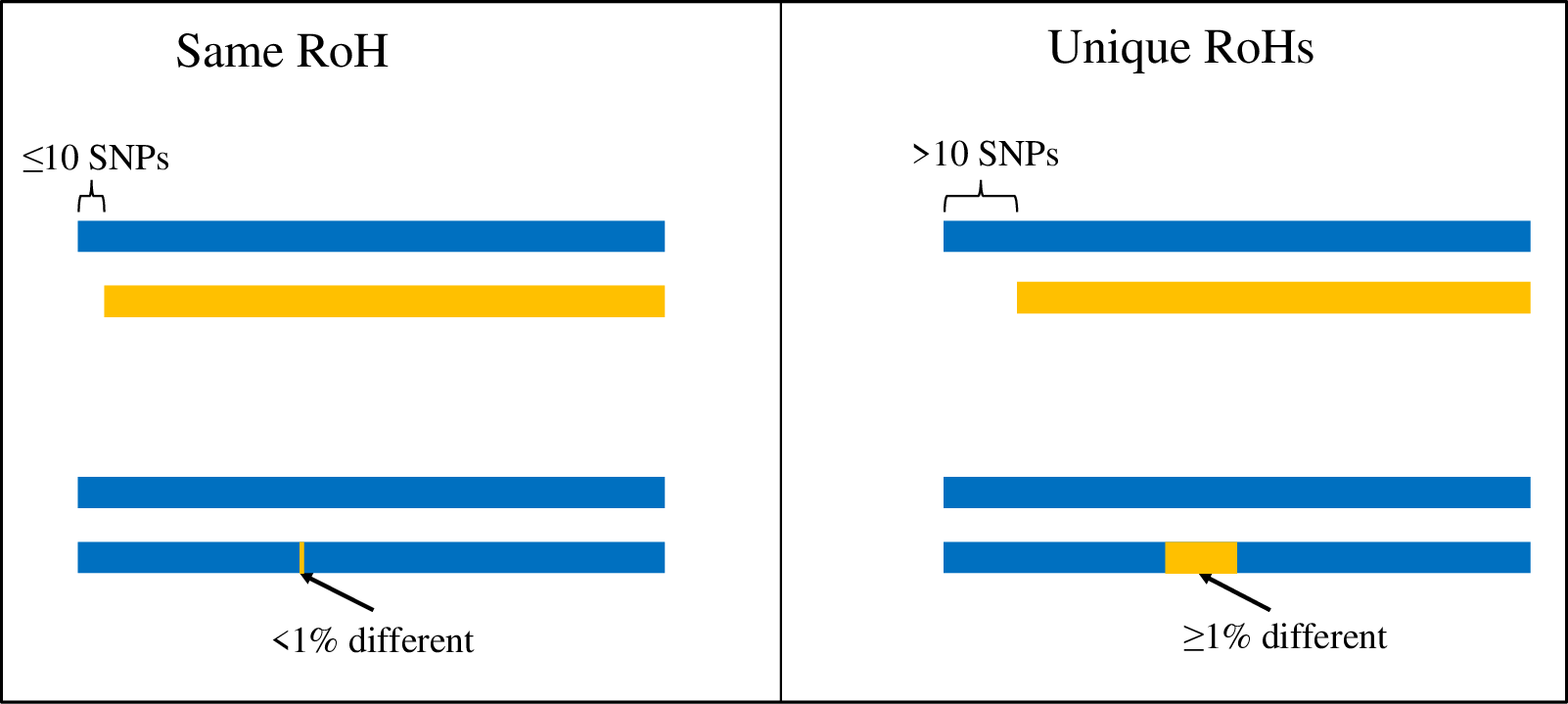

Supplement: S8 Fig — Two runs of homozygosity were considered identical by descent (IBD) if they were 99% identical and had ten or fewer non-overlapping homozygous SNPs. That is, a run of homozygosity in one sample was considered identical to a run of homozygosity in another if it shared 99% sequence similarity and were of similar lengths, defined as ten or fewer non-overlapping homozygous SNPs on either end. Relaxing these assumptions did not qualitatively alter the results nor the conclusions. (TIF) [file pntd.0007392.s008.tif]

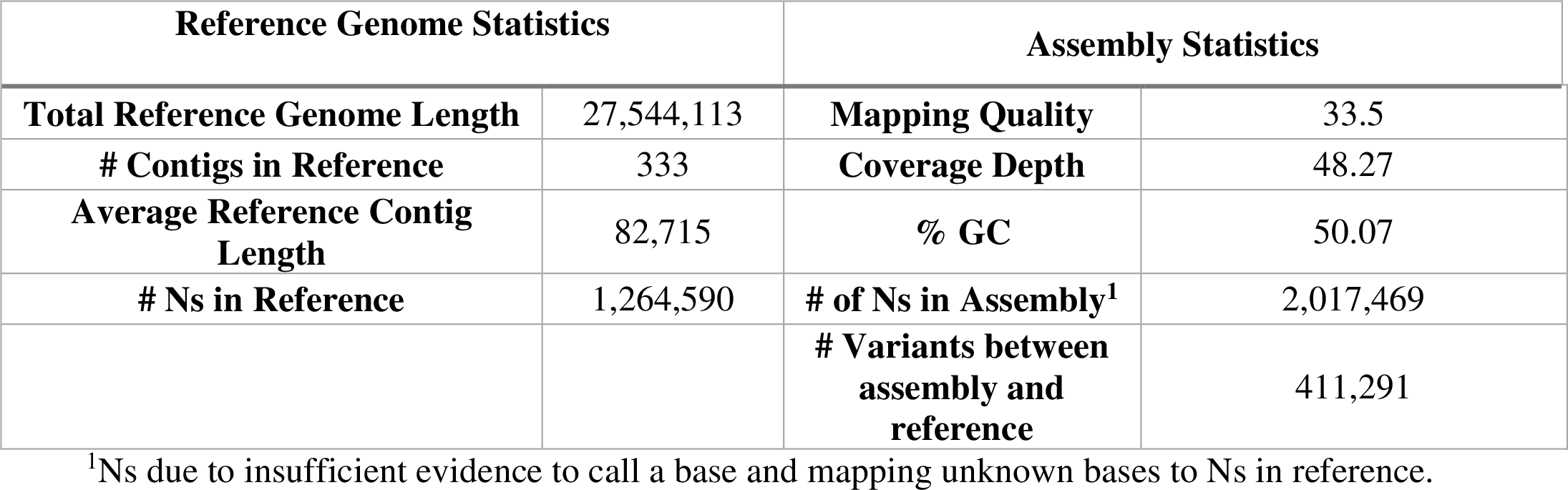

Supplement: S2 Table — (TIF) [file pntd.0007392.s010.tif]
