# Supplementary material for: Sexual reproduction in a natural Trypanosoma cruzi population
Source: PLoS Negl Trop Dis. 2019 May 20;13(5):e0007392. doi: 10.1371/journal.pntd.0007392 (PMC6544315; doi:10.1371/journal.pntd.0007392)
Supplement: S1 Table — (PDF) [file pntd.0007392.s009.pdf]

Sample ID	District	Block	Year Collected	SRA Accession Number
TC125	Bustamante	1	2012	SRS4304930
TC126	Bustamante	1	2012	SRS4304931
TC119	Cayma	1	2011	SRS4304925
TC122	Cayma	2	2011	SRS4304927
TC135	Huanca	N/A	2013	SRS4304983
TC141	Huanca	N/A	2015	SRS4304887
TC142	Huanca	N/A	2015	SRS4304884
TC143	Huanca	N/A	2015	SRS4304885
TC144	Huanca	N/A	2015	SRS4304882
TC145	Huanca	N/A	2015	SRS4304883
TC146	Huanca	N/A	2015	SRS4304890
TC148	Huanca	N/A	2015	SRS4304907
TC149	Huanca	N/A	2015	SRS4304906
TC150	Huanca	N/A	2015	SRS4304909
TC151	Huanca	N/A	2015	SRS4304908
TC152	Huanca	N/A	2015	SRS4304903
TC001	La Joya	4	2008	SRS4304935
TC002	La Joya	4	2008	SRS4304934
TC003	La Joya	5	2008	SRS4304939
TC004	La Joya	6	2008	SRS4304938
TC014	La Joya	3	2008	SRS4304936
TC015	La Joya	7	2008	SRS4304941
TC016	La Joya	4	2008	SRS4304940
TC019	La Joya	6	2008	SRS4304959
TC020	La Joya	4	2008	SRS4304958
TC022	La Joya	2	2008	SRS4304956
TC023	La Joya	9	2008	SRS4304955
TC026	La Joya	4	2008	SRS4304954
TC027	La Joya	6	2008	SRS4304953
TC029	La Joya	6	2008	SRS4304952
TC031	La Joya	9	2008	SRS4304961
TC033	La Joya	6	2008	SRS4304960
TC034	La Joya	8	2008	SRS4304946
TC035	La Joya	9	2008	SRS4304947
TC036	La Joya	9	2008	SRS4304944

TC037	La Joya	6	2008	SRS4304945
TC038	La Joya	9	2008	SRS4304950
TC039	La Joya	9	2008	SRS4304951
TC040	La Joya	1	2008	SRS4304948
TC129	La Joya	1	2013	SRS4304989
TC130	La Joya	1	2013	SRS4304988
TC131	La Joya	1	2013	SRS4304987
TC147	La Joya	1	2015	SRS4304891
TC155	La Joya	1	2015	SRS4304904
TC010	Mariano Melgar	5	2008	SRS4304937
TC041	Mariano Melgar	5	2010	SRS4304942
TC042	Mariano Melgar	2	2010	SRS4304943
TC043	Mariano Melgar	2	2010	SRS4304914
TC044	Mariano Melgar	6	2010	SRS4304916
TC045	Mariano Melgar	2	2010	SRS4304919
TC046	Mariano Melgar	7	2010	SRS4304918
TC047	Mariano Melgar	7	2010	SRS4304921
TC048	Mariano Melgar	7	2010	SRS4304920
TC049	Mariano Melgar	5	2010	SRS4304913
TC055	Mariano Melgar	6	2010	SRS4304894
TC061	Mariano Melgar	2	2010	SRS4304874
TC064	Mariano Melgar	4	2010	SRS4304878
TC065	Mariano Melgar	7	2011	SRS4304860
TC068	Mariano Melgar	12	2011	SRS4304881
TC069	Mariano Melgar	12	2011	SRS4304868
TC070	Mariano Melgar	2	2011	SRS4304869
TC071	Mariano Melgar	1	2011	SRS4304866
TC072	Mariano Melgar	3	2011	SRS4304867
TC073	Mariano Melgar	2	2011	SRS4304864
TC074	Mariano Melgar	3	2011	SRS4304865
TC075	Mariano Melgar	2	2011	SRS4304862
TC076	Mariano Melgar	5	2011	SRS4304863
TC077	Mariano Melgar	5	2011	SRS4304870
TC078	Mariano Melgar	5	2011	SRS4304871
TC079	Mariano Melgar	2	2011	SRS4304854
TC080	Mariano Melgar	5	2011	SRS4304853
TC081	Mariano Melgar	7	2011	SRS4304856
TC082	Mariano Melgar	7	2011	SRS4304855
TC083	Mariano Melgar	7	2011	SRS4304850

TC084	Mariano Melgar	7	2011	SRS4304849
TC085	Mariano Melgar	7	2011	SRS4304852
TC086	Mariano Melgar	7	2011	SRS4304851
TC088	Mariano Melgar	6	2011	SRS4304859
TC089	Mariano Melgar	7	2011	SRS4304858
TC090	Mariano Melgar	6	2011	SRS4304972
TC091	Mariano Melgar	7	2011	SRS4304973
TC092	Mariano Melgar	7	2011	SRS4304974
TC095	Mariano Melgar	12	2011	SRS4304975
TC097	Mariano Melgar	6	2011	SRS4304976
TC098	Mariano Melgar	8	2011	SRS4304977
TC099	Mariano Melgar	6	2011	SRS4304980
TC100	Mariano Melgar	9	2011	SRS4304861
TC101	Mariano Melgar	11	2011	SRS4304978
TC102	Mariano Melgar	6	2011	SRS4304979
TC103	Mariano Melgar	7	2011	SRS4304971
TC104	Mariano Melgar	5	2011	SRS4304970
TC105	Mariano Melgar	3	2011	SRS4304969
TC107	Mariano Melgar	6	2011	SRS4304967
TC108	Mariano Melgar	11	2011	SRS4304966
TC110	Mariano Melgar	10	2011	SRS4304965
TC111	Mariano Melgar	5	2011	SRS4304964
TC112	Mariano Melgar	5	2011	SRS4304963
TC115	Mariano Melgar	1	2011	SRS4304923
TC116	Mariano Melgar	5	2011	SRS4304924
TC124	Mariano Melgar	7	2012	SRS4304929
TC051	Miraflores	1	2010	SRS4304898
TC132	Miraflores	3	2013	SRS4304986
TC133	Miraflores	2	2013	SRS4304985
TC134	Miraflores	2	2013	SRS4304984
TC136	Miraflores	4	2014	SRS4304982
TC137	Miraflores	4	2014	SRS4304981
TC067	Sachaca	1	2011	SRS4304857
TC106	Sachaca	1	2011	SRS4304968
TC113	Sachaca	2	2011	SRS4304962
TC114	Sachaca	2	2011	SRS4304922
TC123	Sachaca	1	2012	SRS4304928
TC053	Tiabaya	1	2010	SRS4304900
TC057	Tiabaya	1	2010	SRS4304896

TC058	Tiabaya	1	2010	SRS4304897
TC127	Tiabaya	5	2012	SRS4304990
TC139	Tiabaya	4	2015	SRS4304889
TC140	Tiabaya	2	2015	SRS4304886
TC153	Tiabaya	3	2015	SRS4304902
TC154	Tiabaya	3	2015	SRS4304905
TC156	Tiabaya	3	2015	SRS4304911
TC063	Uchumayo	1	2010	SRS4304879
TC120	Uchumayo	1	2011	SRS4304926
TC138	Vitor	1	2013	SRS4304888
