# Supplementary material for: Sexual reproduction in a natural Trypanosoma cruzi population
Source: PLoS Negl Trop Dis. 2019 May 20;13(5):e0007392. doi: 10.1371/journal.pntd.0007392 (PMC6544315; doi:10.1371/journal.pntd.0007392)
Supplement: S1 Text — (DOCX) [file pntd.0007392.s011.docx]

*Phylogenetic and Population Genetic analyses*

Geographic clustering of the 56 isolates collected in Mariano Melgar was assessed using 100 independent iterations of ADMIXTURE [1] for each number of genetic clusters (K, ranging from 2 to 8) assuming linkage disequilibrium until the log-likelihood increased by less than ε=10^-4^ between iterations. The optimal number of clusters was estimated to be 4 by the cross-validation score averaged across 100 iterations (Fig S4A). The optimal alignment of the 100 iterations was calculated using CLUMPP [2].

An alignment was generated using presence/absence for each of the 474 recombination events re-coded as G/A for presence/absence, respectively. The phylogenetic relationships among the 123 *T. cruzi* isolates were then inferred by reconstructing the maximum likelihood (ML) phylogenetic tree using MEGA6 [3] implementing a Tamura-Nei substitution model with uniform rates and 1000 bootstrap replicates. The tree was visualized in FigTree (available at <http://beast.bio.ed.ac.uk>).

*Determining the best reference genome*

Bowtie2 [4] was used to assemble genomic reads from the 123 Arequipan *T. cruzi* isolates to both the TcJR and the Silvio (gi|225217165|gb|FJ203996.1) maxicircle reference sequences obtained from NCBI [5]. Duplicate reads were removed from the assembly using Picard’s MarkDuplicates functionality [6]. The assembly had an average depth of >600X across all isolates. Consensus sequences were determined using VarScan [7] ensuring highly-confident base calls by requiring a 60% match to call each SNP.

An alignment that included 28 genetically distinct Arequipan *T. cruzi* isolates aligned to Silvio, the same 28 genomes aligned to TcJRcl4, the Silvio reference sequence (gi|225217165|gb|FJ203996.1|), the Esmeraldo reference sequence (gi|85718082|gb|DQ343646.1), and the CL Brener reference sequence (gi|85718081|gb|DQ343645.1) was created using MUSCLE as implemented in MEGA7 [3]. The ends were trimmed so that all sequences started and ended on the same nucleotide, resulting in a final alignment of 15382bp. A neighbor-joining phylogenetic tree was reconstructed using MEGA7.

*Verifying Recombinant Regions*

Recombination Analysis Tool (RAT) was used to verify recombinant regions [8]. Phased chromosomes were approximated by creating two fasta files for each genome, each containing all polymorphic loci, but differentiated by calling heterozygous sites one base or another in each file. For example, A/G heterozygous sites were called A in one file and G in the other. Recombinant regions were assessed using RAT with a 500bp window size sliding in 200bp increments. Caveats to using this method include: (1) the lack of truly phased genomes and (2) when using whole contigs, due to the close phylogenetic relationship among these samples, the small number of polymorphisms in any genomic window are insufficient for recombination software to identify evidence of recombination. Evidence of recombination may only be apparent when conserved sites are removed, which results in a sequence fundamentally different from a whole chromosome. Nevertheless, RAT did identify many recombinant regions (Supporting Information S1) including all of those we identified. For example, Fig 3 shows a recombination event in TC004 starting near position 42,237 on contig KB222892.1, which corresponds to locus 11,854 in the fasta file used. In the windows containing this recombinant region, which extends to the end of the contig, RAT identified recombination events with most sequences, excluding the other samples in La Joya that we identified as containing the same recombinant region.

**Literature Cited**

1. Alexander DH, Novembre J, Lange K. Fast model-based estimation of ancestry in unrelated individuals. Genome Research. 2009;19: 1655–1664.

2. Jakobsson M, Rosenberg NA. CLUMPP: a cluster matching and permutation program for dealing with label switching and multimodality in analysis of population structure. Bioinformatics. 2007;23: 1801–1806.

3. Kumar S, Stecher G, Tamura K. MEGA7: Molecular Evolutionary Genetics Analysis Version 7.0 for Bigger Datasets. Molecular Biology and Evolution. 2016;33: 1870–1874.

4. Langmead B, Salzberg SL. Fast gapped-read alignment with Bowtie 2. Nat Methods. 2012;9: 357–359.

5. Geer LY, Marchler-Bauer A, Geer RC, Han L, He J, He S, et al. The NCBI BioSystems database. Nucleic Acids Research. Oxford University Press; 2010;38: D492–D496.

6. McKenna A, Hanna M, Banks E, Sivachenko A, Cibulskis K, Kernytsky A, et al. The Genome Analysis Toolkit: A MapReduce framework for analyzing next-generation DNA sequencing data. Genome Research. 2010;20: 1297–1303.

7. Koboldt DC, Chen K, Wylie T, Larson DE, McLellan MD, Mardis ER, et al. VarScan: variant detection in massively parallel sequencing of individual and pooled samples. Bioinformatics. Oxford University Press; 2009;25: 2283–2285.

8. Etherington GJ, Dicks J, Roberts IN. Recombination Analysis Tool (RAT): a program for the high-throughput detection of recombination. Bioinformatics. Oxford University Press; 2004;21: 278–281.
